# Molecular Details of the Frataxin–Scaffold Interaction during Mitochondrial Fe–S Cluster Assembly

**DOI:** 10.3390/ijms22116006

**Published:** 2021-06-02

**Authors:** Courtney J. Campbell, Ashley E. Pall, Akshata R. Naik, Lindsey N. Thompson, Timothy L. Stemmler

**Affiliations:** Department of Pharmaceutical Sciences, Wayne State University, Detroit, MI 48201, USA; courtneycampbell3@wayne.edu (C.J.C.); pall.ashley@wayne.edu (A.E.P.); anaik@med.wayne.edu (A.R.N.); lnico@med.wayne.edu (L.N.T.)

**Keywords:** frataxin, Fe-S cluster biosynthesis, ISC machinery

## Abstract

Iron–sulfur clusters are essential to almost every life form and utilized for their unique structural and redox-targeted activities within cells during many cellular pathways. Although there are three different Fe–S cluster assembly pathways in prokaryotes (the NIF, SUF and ISC pathways) and two in eukaryotes (CIA and ISC pathways), the iron–sulfur cluster (ISC) pathway serves as the central mechanism for providing 2Fe–2S clusters, directly and indirectly, throughout the entire cell in eukaryotes. Proteins central to the eukaryotic ISC cluster assembly complex include the cysteine desulfurase, a cysteine desulfurase accessory protein, the acyl carrier protein, the scaffold protein and frataxin (in humans, **N**FS1, **I**SD11, **A**CP, **I**SCU and **F**XN, respectively). Recent molecular details of this complex (labeled **NIAUF** from the first letter from each ISC protein outlined earlier), which exists as a dimeric pentamer, have provided real structural insight into how these partner proteins arrange themselves around the cysteine desulfurase, the core dimer of the (**NIAUF**)_2_ complex. In this review, we focus on both frataxin and the scaffold within the human, fly and yeast model systems to provide a better understanding of the biophysical characteristics of each protein alone and within the FXN/ISCU complex as it exists within the larger **NIAUF** construct. These details support a complex dynamic interaction between the FXN and ISCU proteins when both are part of the **NIAUF** complex and this provides additional insight into the coordinated mechanism of Fe–S cluster assembly.

## 1. Introduction

Iron–sulfur (Fe–S) clusters are inorganic cofactors that are essential for cell viability in almost every life form [1]. Constructed of ferrous/ferric iron and sulfide, these essential cofactors play an active role in the regulation and operation of many biochemical pathways [2]. Essential pathways that utilize Fe–S clusters include, but are not limited to, cellular respiration and ATP production, as well as several aspects related to cell genetics, including DNA synthesis, damage recognition and repair [3]. The redox and structural versatility inherent in Fe–S clusters allows them to actively participate in targeted activities that include electron transfer and chemical activation reactions, provide biomolecular structural integrity, and it allows them to be used as sensors to gauge environmental iron or oxidation levels in cells and cellular compartments [4]. In prokaryotes, the complexity of Fe–S clusters can vary. However, eukaryotes typically utilize [2Fe–2S] and [4Fe–4S] clusters as biological cofactors [5]. Regardless of the structure, the ubiquitous presence of Fe–S clusters in all of biology highlights their importance in relation to cell viability.

Both the existence and prevalence of Fe–S clusters in nature are a direct result of the ability of iron and sulfur to self-assemble into clusters under anaerobic conditions [6]. During early anaerobic conditions in the Earth’s atmosphere, the prevalence of iron and sulfur in the environment and ease of their ability to self-assemble led to Fe–S clusters becoming incorporated into evolving biochemical pathways within early cells [7]. When the Earth shifted to an aerobic environment, several challenges appeared involving both Fe–S cluster bioassembly and their utilization, since these cofactors can easily be oxidized, becoming structurally compromised. Nature responded by evolving tightly controlled enzymatic pathways that protect Fe–S clusters during their assembly; the complexity of these biosynthetic pathways extended to downstream delivery events too, all of which are tightly regulated. Prokaryotes utilize three different bioassembly pathways to produce Fe–S clusters, including the nitrogen fixation pathway (NIF), the sulfur mobilization pathway (SUF) and the iron–sulfur cluster (ISC) assembly pathway [8,9,10]. Most prokaryotes utilize either the SUF or ISC pathways, but utilization of multiple pathways is often observed. In eukaryotes, proteins within the mitochondrially localized iron–sulfur cluster (ISC) pathway provide for the general need of Fe–S clusters within this organelle, as numerous essential Fe–S cluster-containing proteins operate within this space [1]. The ISC pathway also provides an unknown activated sulfur compound that is essential for the second eukaryotic pathway, the cytosolic iron–sulfur cluster assembly (CIA) pathway. The CIA pathway produces Fe–S clusters utilized outside the mitochondria [1]. While having multiple independent assembly pathways in prokaryotes provides these cells with the flexibility to assembly clusters under different environmental conditions, a complete reliance on the mitochondrial ISC pathway in eukaryotes to directly or indirectly assist in producing Fe–S clusters highlights the extreme importance of this pathway. It is, therefore, not surprising that several human diseases related to deficiencies in the ISC pathway’s normal activity (Friedreich’s ataxia (FRDA) and ISCU myopathy are just two diseases in this area) exist; the clinical importance for understanding ISC protein functions in these diseases is, therefore, obvious [11].

**Figure 1:**
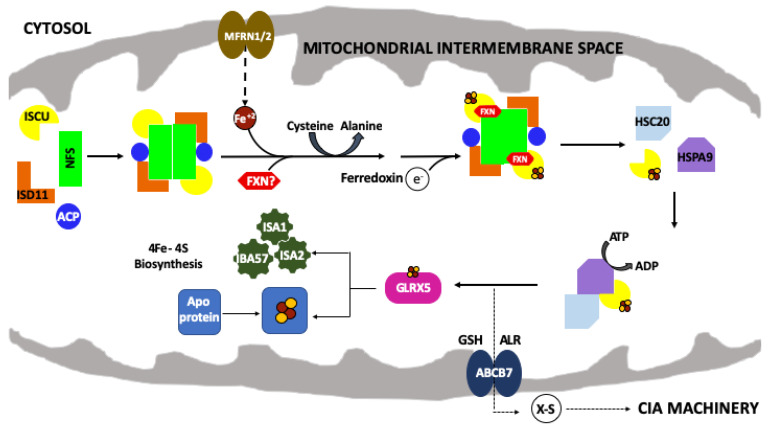
The mitochondrial iron–sulfur cluster (ISC) assembly machinery. Schematic of the de novo mitochondrial Fe–S cluster biosynthetic pathway. Iron (Fe^+2^) is imported via mitoferrin (MFRN1/2) while cysteine desulfurase (NFS1) provides sulfur, from *L*-cysteine, in the form of a persulfide (-SSH). ISD11 and ACP stabilize NFS1. The 2Fe–2S cluster is formed on the scaffold protein (ISCU) and ferredoxin (FDX2) provides the electron required for this process. FXN promotes NFS1 activity and Fe loading of ISCU. HSC20, HSPA9 and GLRX5 receive the 2Fe–2S cluster from the ISC complex and promote downstream delivery. The conversion of 2Fe–2S to 4Fe–4S is still uncharacterized but involves a complex of ISA1, ISA2 and IBA57 proteins. The 2Fe–2S cluster is exported out of the mitochondria as an unknown sulfur-containing moiety (X-S) via the ATP binding cassette (ABCB7).

Housed within the mitochondrial matrix, proteins that are part of the ISC assembly complex utilize ionized iron and sulfur to produce 2Fe–2S clusters [12]. At the core of the ISC multiprotein complex (summarized in [3,13,14]) is the cysteine desulfurase enzyme (NFS1 in humans, Table 1), a pyridoxal phosphate (PLP)-containing enzyme that provides sulfur for Fe–S cluster assembly by cleaving this atom from the side chain of the substrate *L-*Cysteine and storing it in the form of a persulfide (Figure 1). In eukaryotes, NFS1 orthologs require an accessory protein (ISD11 in humans) to both activate and stabilize the cysteine desulfurase. An additional protein, the acyl-carrier protein (ACP1 in humans) closely interacts with ISD11 to form the **N**FS1/**I**SD11/**A**CP1 (**NIA**) protein complex. The exact function of ACP1 has not yet been determined. However, its presence may suggest a correlation between fatty acid biosynthesis and Fe–S assembly [15]. The scaffold protein (**I**SCU2 in humans, or as generalized ISCU) receives persulfide sulfur from the **NIA** complex as well as Fe(II) in a yet identified manner to perform 2Fe–2S cluster assembly. Frataxin (**F**XN in humans) is an iron-binding protein that was originally proposed to be used for Fe storage or as the Fe chaperone for the ISC pathway; however, the chaperone function has lost traction recently and frataxin has been confirmed to serve as a modulator for **NIA** complex activity and for sulfide delivery to ISCU. When two copies of each protein combine within the macromolecular structure, these five proteins make up the **NIAUF** complex that exists as a stable pentameric dimer ((**NIAUF**)_2_, referred to simply as **NIAUF** for the remaining of the review). Electrons are provided by a mitochondrial ferredoxin (FD**X**2 in humans) to form the overall **NIAUFX** complex, which can assemble 2Fe–2S clusters that are transferred to recipient ISCU protein partners for use in events downstream of cluster assembly.

**Figure 2:**
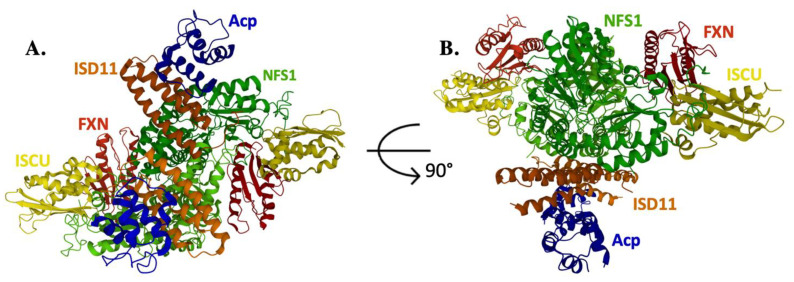
Crystal structure of the human NIAUF complex. (**A**) The dimeric pentamer of the ISC assembly complex has NFS1 as the central linker that forms the dimeric interface. (**B**) ISC assembly complex rotated 90 degrees. NFS1 is represented in green, ISD11 is represented in orange, Acp (bacterial) is represented in blue, ISCU is represented in yellow and FXN is represented in red. PDB ID: 6NZU.

After decades of biochemical and genetic analysis, a compelling structural basis for understanding how the **NIAUF** complex functions came from a series of crystallographic, electron microscopy and NMR structural reports [16,17,18,19]. The initial structure of the human **NI** complex core confirmed the surprising presence of *E. coli* **a**cp, which serendipitously bound to the NFS1/ISD11 complex as a result of the bacterial overexpression system; this initial **NIa** complex existed in the unique “open” conformation, with the dimeric core of the (**NIa**)_2_ structure centered around the two ISD11 proteins at the core dimer interface [18]. This interesting initial structure contrasted with the bacterial cysteine desulfurase dimer observed in a previous bacterial **NU** report [20]; however, the accessory protein Isd11 is not expressed in prokaryotes. The **NIa** structure was followed by a second report both with and without ISCU and divalent metal [16]. This second report, coupled with single angle x-ray scattering (SAXS) analysis, showed a “closed” conformation of the (**NIaU**)_2_ complex, where the dimeric core was constructed by the two NFS1 molecules. This closed conformation was in close agreement to what was observed in the next structural reports using a combined mass spec/SAXS cross linking study, and the closed conformation was used to position FXN binding to form the (**NIaUF**) complex structure seen in the SAXS [17]. Recently, the human (**NIAUF**)_2_ pentameric dimer structure was determined at high resolution using cryo-electron microscopy [19]; this structure also exists in the closed conformation (Figure 2). Finally, a separate detailed interaction of the human ACP-ISD11 interface was also been reported [21]. In nearly every case involving ISCU (except the apo-NIaU structure in [16]), the scaffold attached in the complex contained zinc bound at the active site. Zinc was known to stabilize the protein’s fold in several early scaffold only structures [22,23,24], and it does so by binding to active site cysteine sulfur ligands. In solution, the scaffold has been shown to exist in both structured and dynamic states, depending on the presence or absence of metal, respectively [25]. In the **NIAUF** cryo-EM structure, FXN is orientated with the protein’s β-sheet surface, a region reported earlier from pulldown and spectroscopic studies to be the ISCU binding site, positioned to directly interact with the scaffold [19]. This FXN orientation places the protein’s α-helix 1 Fe-binding site at a position to point away from ISCU and towards NFS1; the appearance of this FXN orientation reduced support that frataxin’s α-helix 1 Fe-binding site is involved in the transfer of iron to the scaffold. Interestingly, the observation that the **NIAUF** complex can exist in two dramatically different core orientations (open and closed), and that ISCU exists in two folded states (structured and dynamic), suggests that molecular dynamics likely play an important role in **NIAUF** function.

In the **NIAUF** complex, NFS1 is central to the complex’s structural core, and pivotal to the Fe–S cluster assembly mechanism, as it provides the activated sulfur required for cofactor formation [19]. Given its high importance, the interactions between the other ISC proteins, specifically FXN and ISCU, are at times viewed secondary in relation to their association to the cysteine desulfurase. However, the initial yeast in vivo pulldown assays were first identified to show the scaffold (Isu1) was a primary binding partner to frataxin (Yfh1) [26,27]. These and other biochemical studies indicated Yfh1 interacted with Isu1 utilizing exposed residues on the frataxin β-sheet surface [28]. Early in vitro biophysical spectroscopic studies confirmed that Yfh1 binds to Isu1 in an iron-dependent manner, and that in the presence of Fe(II), Yfh1 and Isu1 bind with at a nanomolar binding affinity [27]. Recent reports using the human orthologs, however, indicated that FXN binds to ISCU in an iron-independent manner and that when frataxin, ferredoxin and iron were combined, they were all directly important for Fe(II) loading of ISCU as part of the **NIAUFX** complex [17]. With regard to scaffold iron loading, the human, fly, yeast and bacterial orthologs were all shown to bind iron at micro to submicromolar affinity at a location on the protein that is distinct from the molecule’s cysteine-rich active site [28,29,30,31]. In Isu1, binding iron and zinc does not alter the binding characteristics of the opposing metal, and Zn loaded Isu1 was shown to be functionally inhibited towards Fe–S cluster assembly when part of the **NIAUF** complex [31]. This is in contrast with recent reports indicating ISCU iron displaces zinc bound to the protein’s active site when FXN is present, and based on this activity it was suggested that Zn loading of ISCU may regulate protein activity and the cluster assembly pathway in vivo [32]. Differences between partner and metal binding interactions indicate much is still needed to learn about the intermolecular interactions within the ISC pathway. Here, we examine the wealth of biochemical reports targeted to understand both FXN and ISCU, alone and in a protein complex. Discussion related to the two protein’s iron-binding characteristics, their binding to the cysteine desulfurase, the frataxin/scaffold binding interface and residues that provide for protein stability are examined in detail in this report. Our goal is to provide an atomic-level survey of what is known about the interaction between these two proteins, and how this data can help provide a more detailed mechanistic picture of their interactions during Fe–S cluster assembly.

## 2. The Frataxin Protein

Frataxin, a small nuclear encoded highly acidic iron-binding protein, is targeted to the mitochondria where it plays an essential role in the eukaryotic ISC bioassembly pathway [33]. FXN deficiency in humans is at the core of the cardio- and neurodegenerative disorder Friedreich’s ataxia, a rare autosomal-recessive genetic disease typically caused by a trinucleotide repeat expansion in the first intron of the FXN gene; the expansion disrupts transcription of the gene and results in FXN deficiency [34]. Phenotypes of the disorder include mitochondrial iron overload, breakdown in both heme and Fe–S cluster biosynthesis and subsequent generation of reactive oxygen species that both kill the cell and lead to health complications for the organism [35,36,37,38,39]. Given its direct link to the mitochondrial ISC pathway, frataxin’s role in Fe–S cluster assembly has been of great interest. However, despite extensive research, the functional role of frataxin within the ISC pathway remains controversial. Originally thought to participate as a ferritin-like aggregate for mitochondrial iron storage [40], it was later shown that frataxin aggregation activity was dispensable [41] and that frataxin monomers, with a micromolar iron-binding affinity, could directly interact with the scaffold in vitro to promote 2Fe–2S cluster assembly [42]; it was, therefore, suggested that FXN could serve as a chaperone that delivers metal to ISCU [43,44,45]. This idea was supported by in vivo pulldown assays showing the scaffold was the primary binding partner to frataxin in yeast cells and genetic surveys linking frataxin as a direct binding partner [27]. Following these reports, several publications then showed apo-frataxin interacts directly with the cysteine desulfurase and regulates the enzyme’s activity [46,47], providing a strong link that frataxin plays a direct role in regulating persulfide production and delivery within the ISC pathway. With the discovery of a yeast Isu1 mutant that can produce Fe–S clusters in the absence of frataxin [48,49], the idea that frataxin played the essential role of delivering the Fe(II) required for assembly to the scaffold as the iron chaperone has now lost favor. However, regarding the possible roles for frataxin, since a free iron pool at concentration of ca. 150 μM exists in the mitochondria, and that the *K*_d_’s for the frataxin orthologs occur in the range between 5 and 50 μM, frataxin is likely iron loaded when in the mitochondrial matrix milieu [42,45,50].

**Figure 3:**
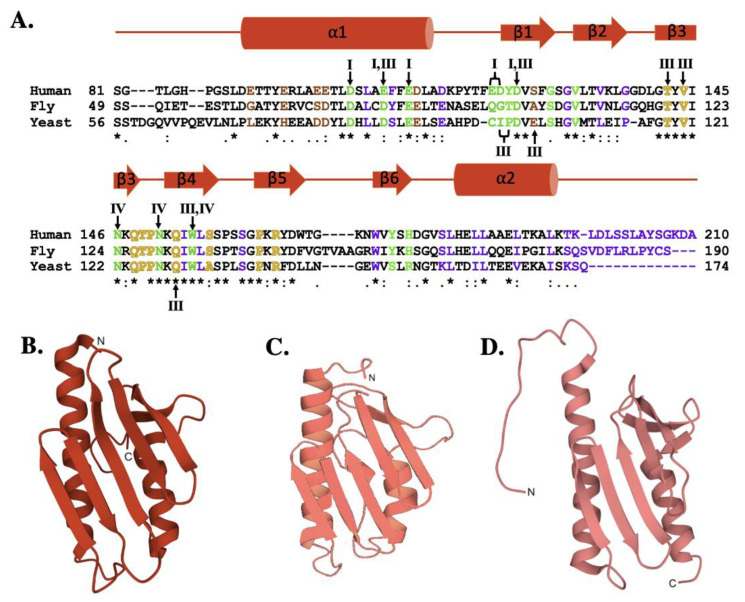
Molecular details of frataxin protein orthologs. (**A**) Sequence homology between human, fly, and yeast frataxin orthologs. Brown colored letters represent iron-binding residues, green indicates residues interacting with cysteine desulfurase, yellow indicates residues involved in ISCU partnering, and purple indicates residues implicated in stability. Likewise, roman numerals are used to represent residues involved in multiple interactions. Roman numeral I represents iron-binding residues, III indicates residues implicated in protein stability, IV indicates residues involved in ISCU partnering. (**B**) Crystal structure of human FXN (PDB ID: 1EKG). (**C**) Crystal structure of fly frataxin modeled using PyMol. (**D**) Crystal structure of yeast frataxin (PDB ID:2GA5).

Frataxin orthologs are highly conserved across species and between kingdoms. The sequence comparison for the human, fly and yeast orthologs (Figure 3A) shows a high degree of homology (42.8% identity between human and fly, 31.7% identity between the human and yeast) that is maintained with the *E. coli* bacterial ortholog (24.5% identity between human and bacterial) [51,52,53]. It is, therefore, not surprising that the secondary structural elements observed between orthologs are also conserved [54]. Regions of functional significance, identified by residue color and Roman numerals in Figure 3A and described in detail below, are also highly conserved in function and molecular location. The tertiary structures of the human, fly, and yeast frataxin orthologs are also highly conserved; with members of this family having an α-β sandwich structural motif fold constructed by two large α-helices on one plane of the proteins helical surface and the second surface constructed by six to six β-strands (Figure 3B–D). When viewed together, these proteins have high structural similarities.

**Figure 4:**
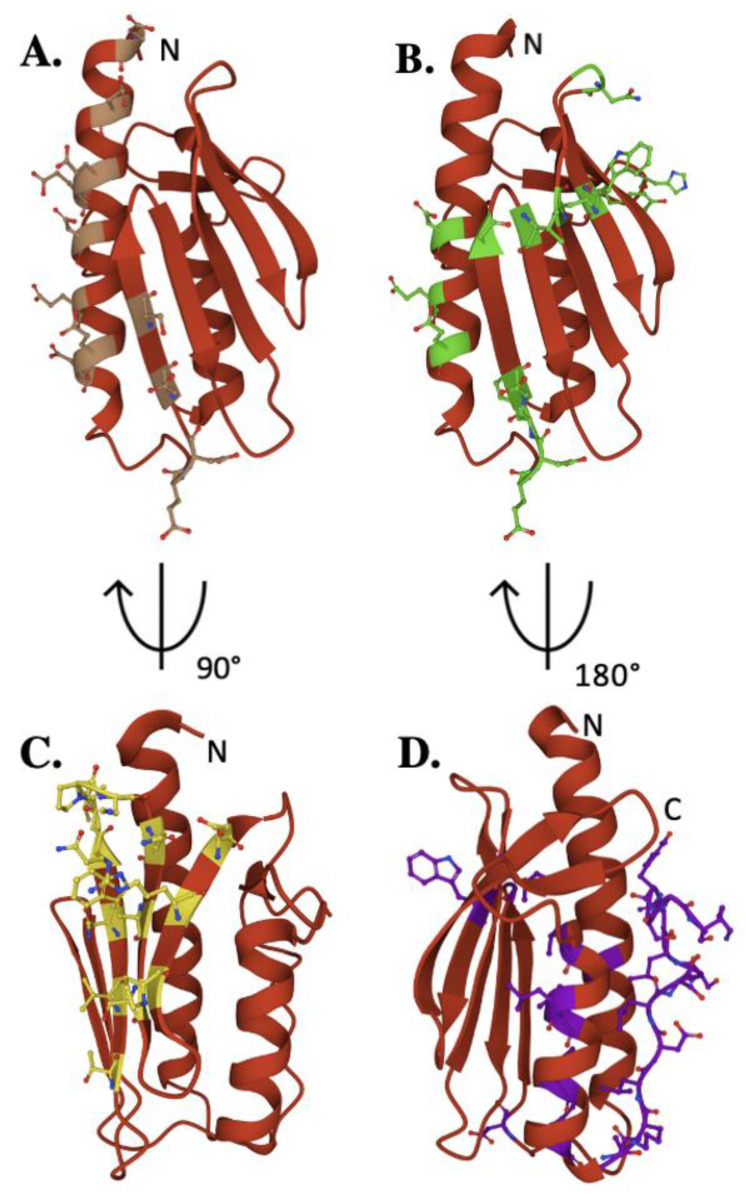
Key residues of biophysical relevance on the human FXN structure. Crystal structure of human FXN with positions of residues for: (**A**) Fe binding (brown), (**B**) cysteine desulfurase binding (green), (**C**) ISCU binding (Yellow) and (**D**) stability (purple).

The iron-binding properties of frataxin orthologs continue to be of interest related to possible functional activities of the protein during Fe–S cluster bioassembly. As shown by NMR chemical shift perturbation assays, FXN’s iron-binding sites are located at the protein’s N-terminal elements, comprised of acidic side chain atoms from highly conserved Asp and Glu residues both in the protein’s α-helix 1/β-strands 1 and 2 regions (Figure 4A) [17,42,55]. Exposed amino acids located at this negatively charged platform provide acidic side chain carboxylate oxygens that can be used for iron binding. A comprehensive mutagenesis survey reported that multi-substituted amino acid derivatives of FXN within this anionic surface could bind iron at reduced metal/protein stoichiometry, indicating this site is fluid in its metal binding abilities [55]. However, mutations involving specific human-equivalent α-helix 1 residues E_92_ and E_96_, separated by one turn of the α-helix, caused a significant decrease in the in vivo ability to produce 2Fe–2S clusters, highlighting the functional importance for these two residues compared to others in this region (Table 2) [55]. Interestingly, human E_92_ and E_96_ are not conserved, in contrast to several other iron-binding residues in this region, suggesting their activity could be unique to the human model system. Iron binding is also not exclusively localized to the α-helix 1 region; frataxin contains a pool of additional acidic ligands capable of participating in binding iron on the β-strands 1 and 2 [55]. Specifically, FXN D122Y mutation lowers the frataxin Fe(II)-binding affinity but does not impact NFS1 binding, so the potential role of this and additional acidic residues within the β-sheet region related to interacting with metal may be important during assembly [56,57]. Assuming frataxin iron binding is in part driven by availability of Fe(II) from the mitochondrial matrix free iron pool, the flexibility of these iron-binding sites provide for a dynamic interaction between protein binding partners in an iron-dependent manner. In addition, residues in this region were also shown by NMR to help drive additional interactions between FXN and both ISCU and NFS1 (Supplementary Table S1) [17]. Finally, Fe(II) bound to frataxin was recently shown to be the source for loading iron onto the **NIAUFX** complex, so it could be these frataxin sites help facilitate ISCU iron loading by directing the metal to the scaffold as it comes from a yet unknown Fe chaperone [55]. While a direct role for frataxin in binding iron remains unclear, the future development of models to elucidate the **NIAUF** reaction mechanism should explore a role of frataxin-bound iron in the process.

Frataxin simultaneously binds regions of both the NFS1 proteins within the cysteine desulfurase dimer, interacting primarily with the C-terminal tail of one NFS1 that fastens the complex to ISCU (Figure 4B, Table 2) [19]. A positively charged Arg-rich region of one promoter of the NSF1 dimer interacts via salt-bridges with the exposed anionic region of frataxin, extending between FXN’s α-helix 1 residues D_104_, E_108_ and E_111_ [17,19]. This interaction is further stabilized by hydrogen bonding between residues in the FXN α-helix 1 to the loop and β-strand 1 residues, where human E_121_, Y_123_ and D_124_ form hydrogen bonds with Arg residues in close structural proximity on NFS1 [19,62]. Mutating FXN D_124_ to Ala results in overall weakend affinity of the protein to the **NIAU** complex. The additional region of FXN binding to NFS1 is located along the β-sheet surface of FXN [19]. In contrast to the bacterial system [72], hydrophobic interactions between W_155_ of FXN and L_386_ of NFS1 orient the protein for direct contact with the catalytic NFS1 cysteine loop responsible for sulfur mobilization [19]. Mutation of W_155_ impairs ISC biosynthesis and triggers cell death, suggesting this FXN residue plays a key functional role in frataxin’s ability to interact with the active site Cys-loop on NFS1 [19]. Finally, the C-terminus of NFS1 wraps around ISCU and is anchored in place by FXN residues N_151_, Y_175_, and H_177_ [19]. Frataxin’s ability to modulate cysteine desulfurase activity is clearly driven by the protein’s ability to selectively interact with NFS1. However, frataxin’s ability to interact with its other major protein partner, ISCU, is likely also equally important.

Despite strong genetic and biochemical data indicating that FXN and ISCU interact in vitro and in vivo, their relative position in the human NIAUF structure indicates that in the complex, they do not interact substantially [16,17,19]. Biochemical data suggests that frataxin β-strands 3, 4 and 5 interact with two regions of ISCU when FXN is bound to NFS1 [17,19]. At the most predominant interface in the structure, the interaction of FXN with ISCU causes displacement within the highly conserved L_131_PPVKLHCSM_140_ region of ISCU, increasing the flexibility of ISCU L_131_PPVK_135_ α-helix (Figure 4C). This allows FXN W_155_ on β-strand 4 to interact with the ISCU active site residues as well as residues on ISCU’s β-strand 3 [19]. An interaction between human FXN W_155_ and ISCU H_137_ could cause a conformational change within the complex which stabilizes the NFS1 Cys-loop, providing sulfur transfer to ISCU. A less prominent interface exists from interactions between FXN N_151_ and the ISCU Ala-loop active site residue C_69_, capable of causing a conformational change within the Ala-loop [19]. Ultimately, FXN associates with NFS1 to stabilize and position the enzyme for persulfide production and sulfur delivery, while concomitantly forming interactions with ISCU that enable the scaffold to receive the activated sulfur and possibly the Fe(II) necessary for Fe–S cluster production.

The C-terminal region (CTR) of the protein plays a key role in the structural stability of human frataxin. The FXN C-terminus is positioned between the α-helices 1 and 2 [70,71]. Here, the CTR functions to shield and stabilize the hydrophobic core of the protein via tertiary contacts extending from the protein’s C-terminal tail (Figure 4D). Truncation of the FXN C-terminus results in significant destabilization of the protein and a severe Friedreich’s ataxia phenotype in eukaryotes [70]. A FXN variant (L_198_R) was recently identified as a CTR-related FRDA causing mutation [71]. Artificially elongated yeast frataxin is more stable than native protein in yeast, likely because of its ability to fold onto itself in a similar manner as seen in the human protein [59]. In a different region, mutations of FXN β-strand 4 residue I_154_ to F affects proximal residues that construct the protein’s hydrophobic core and this results in a significant fold destabilization of the protein [61]. The stability and fold of frataxin in the region of the protein’s iron-binding residues is also highly important; disrupting residues in the α-helix 1 region results in decreased frataxin stability and an inability of the protein to bind iron [34,65,67].

## 3. The Scaffold Protein

**Figure 5:**
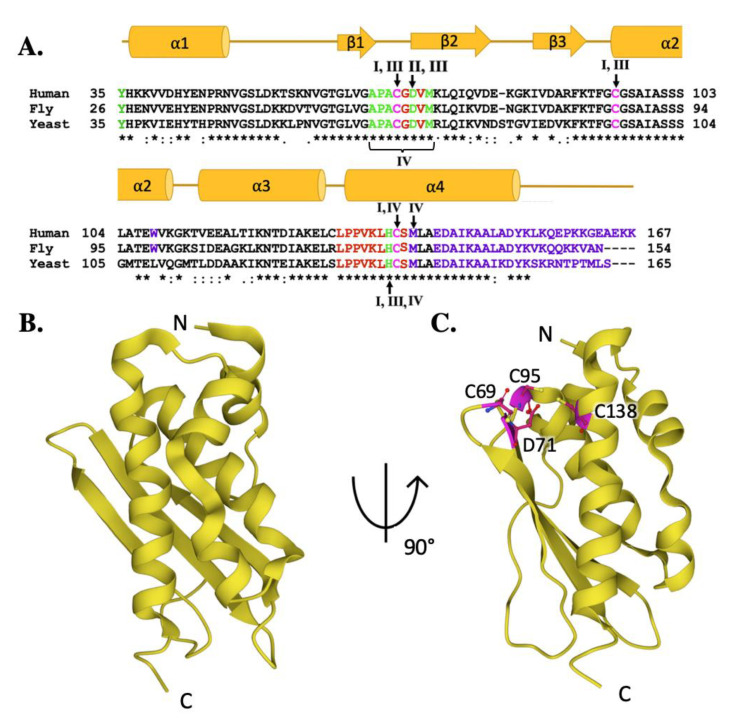
Molecular details of ISCU protein orthologs. (**A**) Sequence homology between human, fly, and yeast scaffold orthologs. Magenta colored letters indicate active site residues, green indicates residues interacting with cysteine desulfurase, red indicates residues involved in frataxin partnering, and purple indicates residues implicated in stability. Roman numerals are used to represent residues involved in multiple interactions. Roman numeral I represents iron-binding residues, II is active site residues, III indicates residues implicated in protein stability and IV indicates resides involved in frataxin partnering. (**B**) Crystal structure of human ISCU (PDB ID: 6NZU). (**C**) Crystal structure of human ISCU (PDB ID: 6NZU) with active site residues highlighted in magenta.

ISCU provides the structural architecture on which mitochondrial iron–sulfur cluster assembly is accomplished. A comparison of the human ISCU amino acid sequence with fly and yeast orthologs (Figure 5A, lower) confirms a high-level of conservation between eukaryotic scaffolds (76.0% identity between human and fly, 61.1% between human and yeast) [52,53]. While only the human ISCU structure has been solved, it is likely the high sequence homology translates into a high conservation of secondary structure when compared to the yeast and fly proteins (Figure 5A, upper). The ISCU tertiary structure, determined in complex with the human cysteine desulfurase, consists of four α-helices which arc around the three anti-parallel β-strands to form a planar platform for the molecule (Figure 5B) [19,20]. The ISCU active site is constructed from three conserved cysteines residues and an aspartic acid residue localized at the solvent-exposed protein edge (Figure 5C) [19,24]. The ISCU active site provides the setting where sulfur is delivered as a persulfide by NFS1 with the assistance of FXN, and once iron is delivered both substrates are used for Fe–S cluster assembly [19]. Inability to express a full-length functional ISCU in humans, as observed in the disease ISCU Myopathy [73], results in Fe–S cluster deficiency, emphasizing the scaffold’s importance in relation to the cluster assembly pathway.

When examining the three human, fly and yeast scaffold sequences (Figure 5A) [51,52,53], it is evident that patterns of residues that completely overlap exist in the proteins primary structure and these regions have, not surprisingly, conserved functional significance (see regions identified in Roman numerals). Biochemical and genetic studies present a unified role for specific residues in these regions in relation to supporting ISCU cluster assembly activity within the **NIAUF** complex. Specific residues of interest include (a) the N-terminal tyrosine (Y_35_), (b) the Ala loop-containing active site cysteine (human C_69_) and aspartic acid (D_71_) residues, (c) the active site cysteine located on α-helix 2 (C_95_), (d) the semi-conserved α-helix 2 tryptophan (W_108_) present in fly and human, but absent in yeast, (e) LPPVK residues at the N-terminus of ISCU α-helix 4 (L_131_-PPV-K_135_), (f) the active site cysteine (C_138_) on α-helix 4, and (g) the entire C-terminal α-helix (P_132_ through Y_153_). The tertiary structure of ISCU, shown in Figure 5B, provides a visual orientation of the residues and their secondary structure outlined in Figure 5A, only in the protein’s 3D configuration, [19] while ISCU active site residues are highlighted in Figure 5C. Despite high sequence homology and likely very similar structures, subtle biochemical differences between these three orthologs have been observed and are worth noting. The fly ISCU ortholog is extremely stable against aggregation and degradation compared to the human and yeast proteins [30]. The fly ISCU ortholog binds Fe(II) at a tighter affinity (sub μM *K*_d_’s) than the human and yeast proteins [30]. The fly ortholog binds as part of the **NIAUF** complex in a more dynamic manner, in relation to binding affinity and faster off rates, but can produce 2Fe–2S clusters at accelerated rates [30]. Despite these differences, all three orthologs (as well as the bacterial protein) have been shown to bind iron at a location that is distinct from the protein’s active site [29]. To better understand both similarities and differences in the solution properties of these three orthologs, a compilation of specific residues genetically and biochemically targeted for their role in iron binding/utilization, generating a binding interface with either cysteine desulfurase or frataxin, and amino acids important for protein stability has been generated (Table 3). Specific residues important for these properties are discussed in more detail below.

**Figure 6:**
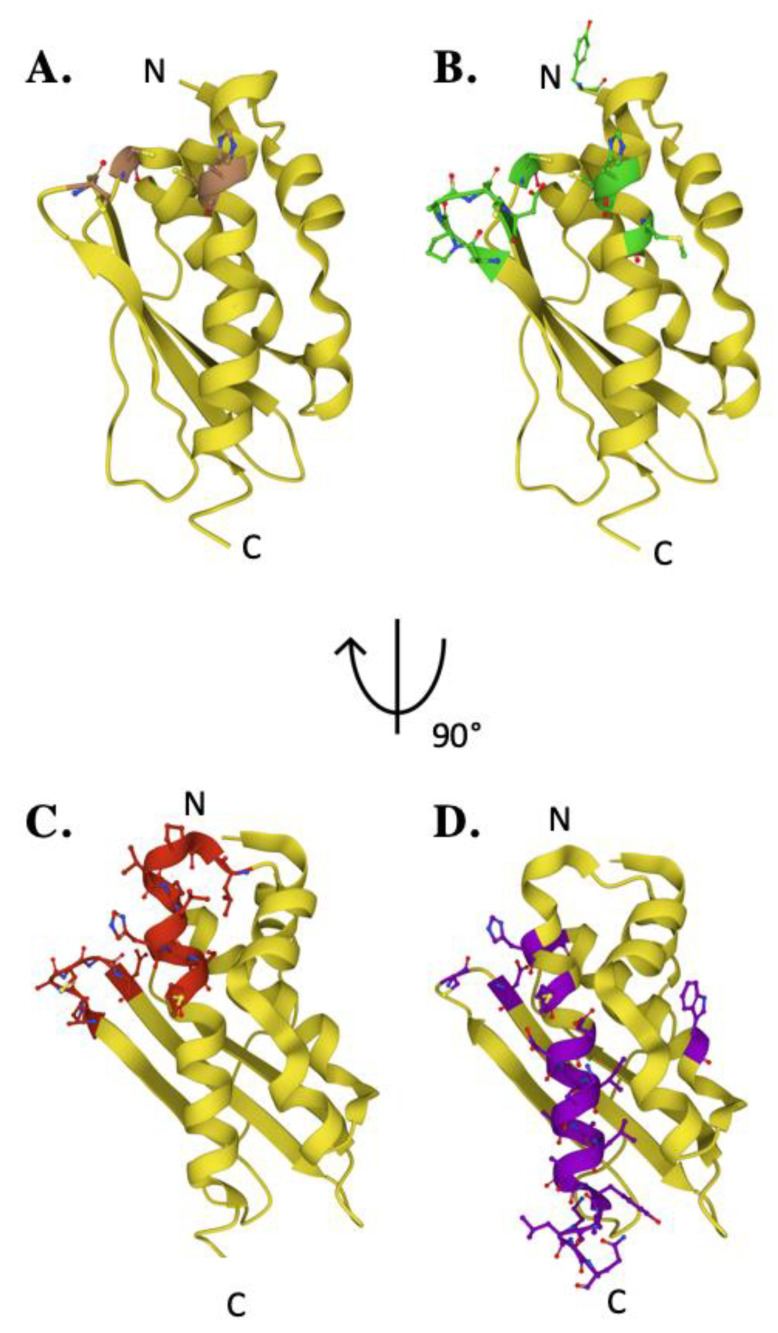
Key residues of biophysical relevance on the human ISCU structure. Crystal structure of human ISCU with positions of residues for: (**A**) Fe binding (brown), (**B**) cysteine desulfurase binding (green), (**C**) FXN binding (red) and (**D**) stability (purple).

Fe–S cluster assembly is accomplished once the iron and sulfur substrates are delivered, and residues on the scaffold orthologs recognized in the solution and cellular studies to participate in metal binding are highlighted in Figure 6A. In the zinc-loaded human ISCU structures, Zn^2+^ binds directly to active site residue side chain atoms from C69, D71, C95 and H137 [16,19]. Zinc binding was shown earlier to stabilize the scaffold fold—as a soft acid, it binds to soft base atoms (sulfur) to form a coordination assembly that likely represents binding of a low-spin Fe(II)–S complex [19,82]. Although a 2Fe–2S cluster-bound structure of human ISCU has not yet been determined, a snapshot of what a 2Fe–2S cluster bound to the scaffold likely looks like is seen in the cluster loaded structure of the ortholog from *Aquifex* *aeolicus* [83]. In the *Aquifex* structure, which exists as a protein trimer, while two of the proteins are in the apo form the third contains a 2Fe–2S cluster bound by two Cys side chain sulfurs at one Fe atom and a Cys and a His side chain sulfur and nitrogen, coordinated to the second iron. A Cys(-S)_3_His(-N)_1_ first atom ligand coordination environment directly attached to the two irons in a 2Fe–2S cluster is consistent with the sulfur/nitrogen ligand coordination atomic ratio observed for a cluster loaded yeast and fly scaffold in solution by X-ray absorption spectroscopy (XAS) [84]. The S_3_N cluster coordination scheme, as compared to a S_4_ environment, is likely beneficial for cluster release since His nitrogen ligation may partially destabilize 2Fe–2S cluster binding to the protein in preparation for transfer during cofactor delivery events following assembly [83].

Despite having some structural information of different scaffold orthologs with zinc and a 2Fe–2S bound, two additional aspects related to the ISCU iron-binding activity need to also be considered. First, as discussed earlier, several publications show that apo-human, fly and yeast scaffolds bind iron with micromolar to sub-micromolar affinity at a site that is “distinct” from the protein’s Cys-rich active site [29,31,32]. Association of the six-coordinate high-spin Fe(II) atom to the scaffold at this distinct site is presumably accomplished utilizing Asp, Glu, and His side chain oxygen/nitrogen atoms as metal binding ligands. Long-range (>2.5 Å) scattering observed in the solution XAS data for these samples confirms that Fe(II) is coordinated to the protein and not just adventitiously associated in solution [29,30,31,80], although a fraction of the six direct iron ligands may come from oxygen due to coordinated water molecules. In the yeast system, iron binding at this distinct site is not affected by zinc coordination at the Cys active site [31]. In yeast, zinc binding to Isu1 within the yeast **NIAUF** complex also renders the system functionally inactive towards 2Fe–2S cluster assembly [31]. This is in contrast to a recent publication indicating FXN binding the Zn-**NIAU** complex promotes iron displacement of ISCU bound zinc and allows ISCU to be fully active towards cluster assembly [19]. Although Fe(II) binding to a site distinct from the active site has been observed by Mössbauer spectroscopy on a mouse apo-ISCU ortholog [32], this iron amounted to only a small percentage of the overall Fe loaded [29]. In the XAS studies, the absence of any Fe–S ligation in the XAS for each of the human, fly and yeast scaffolds suggests that iron bound to this distinct site is the predominant species in these samples. While the physiological relevance of a distinct Fe-binding site on all scaffolds distant from the Cys-rich active site needs to be explored further, these results could have direct implications into the initial steps of the cluster assembly mechanisms for ISCU iron loading. A second consideration that needs to be explored is that in the *Aquifex* scaffold trimer, while one protein in the trimer contains a loaded cluster, the other two protein monomers are in the apo-state [84]. Both apo-molecules have residues in the N-terminal portion of the protein’s C-terminal α-helix that are unwound and dynamic in their structure; this region contains active site residues H_137_ and C_138_, as well as M_140_, the Met residue that suppresses the need for FXN in ISCU driven cluster assembly events when mutated [84]. Assuming ISCU does not bind to the **NIAU** complex coming preloaded with Fe(II), an iron loading event could promote folding of the protein in this region as part of the delivery of metal to the active site residues [31]. In the apo-ISCU structures, as part of the **NIaU** complex, residues in this ISCU C-terminus are already in a structured state, possibly reflecting a structural orientation of the active site that models events after a cluster has already been formed [16]. Interestingly, NMR analysis of scaffold orthologs identified both structured and dynamic forms exist in solution, depending on metal loading state of the biomolecule [25,76,79,85,86]. The structured vs. dynamic fold exchange is certainly also functionally significant in relation to the assembly mechanism and must also be considered.

It is not surprising that NFS1 interacts with ISCU utilizing conserved scaffold residues in close proximity to the active site cysteine (Figure 6B); there is an obvious benefit for this coordination to complete persulfide transfer [19]. The cysteine desulfurase can transfer persulfide directly to ISCU active site C_138_ residue within this orientation, so interactions between these two partner proteins within this region are essential. The ISCU Ala loop (A_66_-C_69_) contacts NFS1 residue (Glu_399_ and Trp_454_) side chains to orient binding in this intermolecular interface; upon binding, residues in the Ala-loop region then undergo a substantial structural change to promote persulfide delivery [16]. When ISCU-equivalent residues L_63_, V_72_, and F_94_ were mutated to alanine in the yeast ortholog, a substantial reduction in binding affinity between Isu1 and Nfs1 was observed during pulldown assays, indicating these three Isu1 residues were likely involved in forming the intermolecular interface for cysteine desulfurase binding to the scaffold [78]. These three nonpolar resides are positioned to be highly exposed within a generally hydrophilic surface of the scaffold, supporting the idea they interact with hydrophobic residues on the cysteine desulfurase to form a favorable intermolecular interface during delivery [78]. Independent studies on the human protein showed that when ISCU-equivalent residues D_71_ and M_140_ were mutated to valine and isoleucine, respectively, the apo-scaffold fold shifted from the dynamic “D” to the structured “S” state [17,86]. Wildtype ISCU can participate in forming a stable **NIAUF** complex, while mutant ISCU is dynamic, indicating the dynamic nature of scaffold residues at these positions may help control the intermolecular stability of the complex when bound to the cysteine desulfurase [86]. In the absence of FXN, the M_140_I ISCU mutant is still capable of receiving persulfide from the **NIA** complex, and the mutant ISCU remains active towards Fe–S cluster assembly [48,49]. This indicates the role of FXN during persulfide catalysis and mobilization can also be bypassed with this mutant, confirming iron delivery to ISCU and activity of the **NIAU** complex can still proceed.

Early reports utilizing yeast pulldown assays linked Yfh1, as well as Nfs1, as primary binding partners to the Isu1 yeast scaffold [87,88]. Genetic and spectroscopic studies indicated that frataxin orthologs interact with ISCU utilizing residues on the frataxin β-sheet surface [19]. The complementary ISCU residues at the FXN binding surface include L_131_-PPVKLHCS-M_140_; this ISCU region was also implicated as a potential binding site for chaperone proteins used during downstream cluster delivery (Figure 6C) [19,78]. In several cases, residues related to NFS1 binding on ISCU are identical to the FXN binding residues on the scaffold. In the human **NIAUF** structure, the distance between the ISCU residue C_69_, which is part of the Ala loop region (Ala_66_–Asp_71_), and FXN N_151_, potential intermolecular hydrogen binding residues, is 7.03 Å (backbone atom to backbone atom) [19]. Based on structural data, FXN binding to the **NIAU** complex induces an ISCU conformational change in this region which promotes an enhanced interaction between cysteine desulfurase and the Zn loaded ISCU [19]. Finally, a possible overlap at this Ala loop site between ferredoxin and FXN to promote electron donation and persulfide transfer interactions, respectively has also been suggested for residues in this ISCU region [78,87].

Several residues have been shown to support the structural and biochemical integrity of the scaffold molecule. The N-terminal tyrosine (human Y_35_) is important for internal stability of the bacterial scaffold (Figure 6D) [77]. This residue is important for stabilizing the interaction between the scaffold and cysteine desulfurase; the bacterial Y_35_-equivalent interacts with bacterial cysteine desulfurase through a hydrogen bond from the Tyr aromatic hydroxyl unit. In bacteria, when this residue was substituted with 19 different amino acids, only the Y_35_F, H and W mutations were functionally equivalent, albeit cells grew at lower growth levels, indicating that aromatic or imidazole ring atoms at this ISCU position are essential for protein stability [77]. In addition to the ISCU’s N-terminal tyrosine, human-equivalent residues C_69_, D_71_ and H_137_ were shown by NMR chemical shift perturbation studies through mutagenesis to be important for maintaining a stable active site structure, indicating they are also essential for protein stability [17,86]. A unique solvent exposed W_108_ and the orientation of this residue’s side chain is also of particular interest. It has been suggested this residue may participate at the ISCU homonuclear dimer interface, serving as a stabilizing factor when two molecules come together [86]. Finally, our lab explored the role of the scaffold C-terminal α-helix residues in relation to both iron binding and towards promoting Fe–S cluster assembly when in the **NIAUF** complex [81]. The C-terminal α-helix is of particular importance in relation to the human disorder ISCU myopathy [73]. While residues in this C-terminal α-helix do not play a role in iron binding, C-terminus deletions reduced the ability of truncated scaffold to participate in cluster assembly, possibly due to the protein’s inability to interact with protein partners [81].

## 4. Frataxin/Scaffold Interactions

Earlier in this report, we evaluated the molecular characteristics of frataxin and scaffold independently to provide a fundamental understanding of the biophysical characteristics of each protein on their own. In this section, we analyze the coordinated interactions observed between these two protein partners to help present a comprehensive dynamic picture of how they operate together before, during, and after Fe–S cluster bioassembly. A clear picture of their relative orientation, presented in the recent **NIAUF** structures, shows the molecular details of how FXN and ISCU interact under controlled conditions (i.e., a structured scaffold protein loaded with Zn^2+^). Comparing that picture to the one obtained from the **NIAU** structure alone frames the ISCU structure under conditions ± being Zn loaded before and/or after an interaction with FXN, and this can be used to frame a discussion regarding how FXN initially interacts with the complex. It is also beneficial, however, to dissect the additional interactions observed to also occur in solution between these protein partners from the in vivo (genetic analysis) and in vitro (biophysical characterization) analysis, since these interactions add to the overall reaction mechanism story. Beginning with the structural description of the ISCU in complex with **NIAU** with and without FXN, we will discuss additional observations that frame a dynamic picture of molecular details during assembly by the **NIAUF** complex.

**Figure 7:**
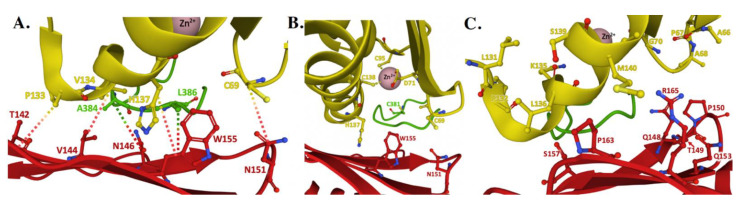
Structure of the human FXN–ISCU interface from the NIAUF crystal structure. (**A**) Residues with direct interaction on FXN (red) and ISCU (yellow). (**B**) Residues at the ISCU zinc-loaded active site with the cysteine loop and catalytic residue C_381_ of NFS1 (green) depicted behind the protein partners. (**C**) Residues at the FXN–ISCU interface whose association are supported by biochemical data. Adapted from PDB ID:6NZU.

Since frataxin is a known scaffold partner, the biophysical characteristics of its binding interaction has been an area of intensive interest. In the yeast model system, Yfh1 in solution binds to Isu1 alone in an iron-dependent manner at nanomolar binding affinity (1:1 stoichiometry at *K*_d_’s of 166 ± 112 and 5 ± 3 nM) [28]. FXN, however, was shown to interact with ISCU in an iron-independent manner at similar affinities [17]. The interaction observed between FXN and Zn-loaded ISCU, as part of the human **NIAUF** structure, provides atomic-level insight into the several hydrophilic and hydrophobic intermolecular interactions between conserved residues from both molecules when the ISCU active site is metal loaded with Zn(II) (Figure 7A). Several intermolecular interactions between FXN and ISCU, established through side chain atom contacts between FXN•••ISCU in the structure, include T_142_•••P_133_, V_144_•••V_134_, W_155_•••N_137_ and N_151_•••C_69._ FXN also makes contacts with NFS1 through respective FXN•••NFS1 interactions at N_146_•••A_384_ and W_155_•••L_386_. In this structural orientation, frataxin interacts directly with Zn(II)-loaded ISCU while also interacting with the NFS1 catalytic Cys-loop; in a combined view, this interaction likely would help facilitate sulfur mobilization for liberating the Cys-loop to promote persulfide loading and delivery to ISCU [19,32]. Under both the **NIAUF** and **NIAU** structures, ISCU is Zn loaded and the protein’s C-terminal α-helix is completely folded, indicating ISCU in these structures is in its structured conformation.

Differences observed in Zn coordination between the **NIAU** and **NIAUF** complex structures indicates frataxin alters the zinc-ligand architecture related to NFS1 and ISCU in the cluster assembly active site region of the scaffold. Biochemically, zinc inhibits NFS1 sulfur mobilization in the absence of frataxin, and based on the **NIAU** structure it does this through direct coordination of Zn(II) to both ISCU active site residues and to the NFS1 catalytic Cys-loop residue C_381_. In this manner, Zn binding obstructs the availability of the NFS1 persulfide transfer Cys while statically constraining the enzyme’s Cys-loop so it would be unable to reposition back into the cysteine desulfurase active site pocket for persulfide attachment. In the **NIAUF** structure, binding of FXN shifts the Zn ligands to being solely ISCU based, liberating the NFS1 C_381_ as a metal ligand and allowing for repositioning of the NFS1 Cys-loop to be available to move, through interactions with FXN N_151_, into the NFS1 active site (Figure 7B). Assuming Zn binding to ISCU is physiologically relevant, these structures provide atomic-level details into how zinc could regulate the ISC pathway by controlling the key active site residues in the ISC protein apparatus, as suggested recently [2].

However, several points related to Zn binding cloud the relevance for a specific role of zinc within the ISC assembly pathway. These include (I) the availability of free zinc in the mitochondrial matrix is extremely limited (measured in the sub-picomolar range [89,90]), so using a low availability free metal to ISCU for regulating this pathway, especially at ISC protein concentrations that are orders of magnitude higher in concentration, needs to be evaluated. A Zn specific chaperone would be required to load zinc metal onto the scaffold if this were a physiologically relevant process. (II) The affinity for ions, as described by the Irving–Williams series, indicates next to copper, zinc ions have the highest affinity for binding of all the biologically relevant divalent metal ions [91]. Having a weaker binding iron ion displace a bound zinc, especially under basic pH conditions within the mitochondrial matrix which would make Zn(II) bind even tighter, seems counterintuitive. A comparison of the Zn(II) and Fe(II) metal binding affinities for the yeast Isu1 scaffold physically showed zinc binds at an order of magnitude tighter metal binding affinity than iron, so again having Fe displace bound Zn is the opposite of what you would expect. (III) While unique to what was seen in the human system, Zn irreversibly inhibits Isu1 cluster assembly activity within the yeast **NIAUF** complex, so a role for zinc in helping activate the pathway is contrary to what we observe [31]. Finally, (IV) the human equivalent of the M_140_I FXN suppressor ISCU mutant remains active in vivo under conditions where FXN is not present to regulate NFS1 activity or disrupt the NFS1 C_384_ ligation to Zn bound to ISCU, and this is contrary to the theory that zinc would play a normal role in regulating the process since it presumably could not be liberated from ISCU in the absence of FXN [48]. We, therefore, conjecture that the structural snapshots observed when zinc is coordinated to ISCU while being extremely important, are more likely relevant for describing a modelled ISCU active site where a single Fe–S unit or a full 2Fe–2S cluster is bound to the scaffold, hence mimicking later events within the Fe–S cluster assembly mechanism. Therefore, examining the wealth of genetic and biophysical results indicating how FXN and ISCU interact in solution that are not consistent to the structural data may provide insight into the early steps within the reaction mechanism.

In the **NIAUF** structure, interface contacts between frataxin and scaffold are dominated by exposed residues on the FXN β-sheet surface and on the ISCU α-helix 4. In the literature, however, there are several additional interactions noted (Table 2 and Table 3) that also have significance related to forming an intermolecular interface between these protein partners. However, these interactions are not consistent with the snapshot presented by the **NIAUF** structures (i.e., at intermolecular distances outside 10 Å in the structure). From the perspective of frataxin, residues identified and marked with respect to the human sequence including Q_148_, T_149_, P_150_, Q_153_ and S_157_ in the β-strand 3-loop-β-strand 4 region along with P_163_ and R_165_ on β-strand 5, all of which are on FXN’s β-sheet surface, have also been implicated as interacting with scaffold orthologs (Figure 7C). In the case of the scaffold orthologs, relevant residues on the human ISCU sequence including A_66_-A_68_ and G_70_ in the β-strand 1-loop-β-strand 2 region as well as α-helix 4 residues L_131_, P_132_, K_135_, L_139_ and M_140_ are reported to make interactions with frataxin in solution. To allow for these interactions to occur, there would need to be a shift in the relative binding orientation between FXN and ISCU from what is reported in the **NIAUF** structure, or an unfolding on the ISCU N-terminal region at α-helix 4, which would make this region flexible enough to interact with FXN in a manner not represented in the current structure. Unfolding of the N-terminal region of α-helix 4 would be consistent with the structural profile for the two apo-scaffold oligomers observed in the *Aquifex* structure.

## 5. Discussion and Health Relevance

From a biochemist’s perspective, this is an exciting time to explore the ISC protein machinery. There are so many exciting labs providing new details related to how the ISC protein complex assembles and functions, and their molecular and atomic details provided by several structural biology groups, helps enrich our knowledge of the pathway. However, this work also raises additional important questions. These questions include, how is iron introduced and used by the ISC machinery for Fe–S cluster assembly? Does ISCU bind as part of the **NIAUF** complex pre-loaded with Fe(II), and if so, does it require a yet unknown iron chaperone to load its metal or is iron be abstracted from the abundant mitochondrial free iron pool [92]? Is ISCU Fe(II) loaded after forming the **NIAU** or **NIAUF** complex, and if so, what is the iron delivery mechanism and does FXN play a role in this process? Related to this question, where is the ISCU Fe(II)-binding site (i.e., does Fe(II) bind directly to the ISCU active site or at an unknown distinct location)? Is ISCU Zn loading a physiologically relevant event possibly used to regulate the ISC pathway, and if so, what is the process for ISCU Zn loading and what is the functional nature of Zn inhibition differences observed between scaffold orthologs? These big-picture questions underscore just some of the important questions that still need to be answered.

**Figure 8:**
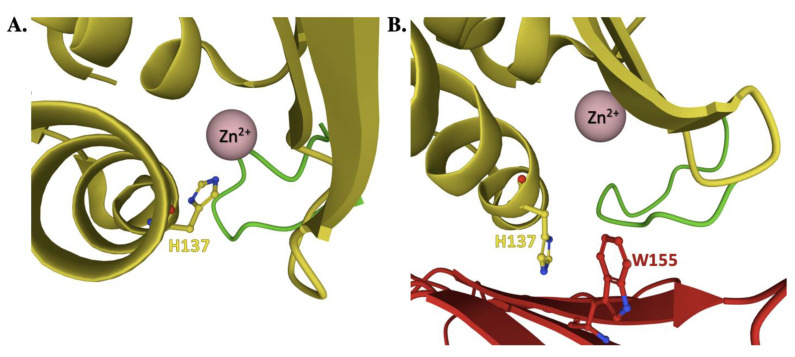
The structural orientation of ISCU residue H_137_ in the Zn-NIAU crystal structure compared to Zn-NIAUF. (**A**) The Zn-loaded NIAU crystal structure (PDB: 5WLW) with the imidazole side chain of human ISCU (yellow) residue H_137_ pointing towards the ISCU active site. (**B**) The Zn-loaded NIAUF structure with the imidazole side chain of human ISCU residue H_137_ projecting away from the ISCU active site to orient parallel with the aromatic plane of human FXN (red) residue W_155_. Adapted from PDB ID:6NZU.

There are also questions at the atomic level that need to be addressed when developing a comprehensive Fe–S cluster reaction mechanism model for protein activity of the ISC pathway. What is the role of the ISCU active site residues in coordinating metal loading and in assembling 2Fe–2S clusters? During assembly, is a single ISCU molecule loaded with one Fe(II) ion, requiring ISCU dimerization for 2Fe–2S cluster assembly, or is a full 2Fe–2S cluster assembled on each ISCU monomer when part of the **NIAUF** complex? Human D_71_ provides a ligand to the Zn(II) bound to ISCU in the **NIAUF** structure, a structure we conjecture more closely mimics a 2Fe–2S loaded scaffold and the site that matches that seen for the cluster loaded *Methanothrix thermoacetophila* scaffold [93]. However, the human H_137_ residue equivalent in the *Aquifex* 2Fe–2S loaded structure serves as a direct cluster binding ligand in this structure, so is this residue utilized to direct Fe(II) ligation when forming the cluster? It is interesting to see that H_137_ exists in a unique structural orientation depending on the presence of FXN. In the apo- and Zn-loaded **NIAU** structures, the residue points towards the ISCU active site. However, upon coordination with frataxin, the imidazole side chain projects away from the ISCU active site to an orientation where it stacks parallel to the aromatic plane of FXN W_155_ (Figure 8). Does this conserved His play a specific role in ISCU iron-loading, in 2Fe–2S cluster assembly, or in a molecular reorganization event for ISCU fold coupled with Fe–S cluster assembly? Finally, is folding in the proximity of the ISCU C-terminal α-helix at conserved residues L_131_-PPVKLHCSM_140_ relevant to early- or late-stage events that occur during Fe–S cluster assembly? All these questions still need to be answered.

Getting answers to these either high-level or narrowly focused questions will help the Fe–S cluster assembly community build a more comprehensive understanding of events that lead to ISC protein complex reactivity. When considerations associated with the FXN–ISCU intermolecular interactions that are not obvious from the current NIAUF complex structures are clarified, or considerations related to the scaffold dynamics (as a monomer or part of the complex) are addressed, we will have a more dynamic model of specific events that allow for the production of these essential Fe-cofactors in eukaryotes.

## Figures and Tables

**Table 1:** Nomenclature of ISC Proteins in the Human, Fly and Yeast Systems.

Protein Name	Human		Fly		Yeast	
	Symbol	MW (kDa)	Symbol	MW (kDa)	Symbol	MW (kDa)
Cysteine desulfurase	NFS1	50.2	dNfs1	51.1	Nfs1	54.5
LYR Motif-containing protein 4	ISD11	10.7	dIsd11	11	Isd11	11.2
Acyl carrier protein	ACP/NDUFAB	17.4	dAcp	17.2	Acp1	13.9
Iron–sulfur cluster assembly scaffold	ISCU	15.3	dIscU	16.7	Isu1	17.8
Frataxin	FXN	23.1	dFh	20.9	Yfh1	19.5

**Table 2:** Notable Frataxin Residues.

Function	Residue	Mutation	Location on Protein	Organism	Hs Equivalent	Reference
Iron Binding	E92	E92A	α1	*Hs*	E92	[55]
	E96	E96A	α1	*Hs*	E96	[55]
	E100	E100A	α1	*Hs*	E100	[55]
	E101	E101A	α1	*Hs*	E101	[55]
	D104	D104A	α1	*Hs*	D104	[55]
	D86	D86A	α1	*Sc*	E108	[58]
	E108	E108A	α1	*Hs*	E108	[17,42]
	E111	E111A	α1	*Hs*	E111	[55,59]
	D112	D112A	α1	*Hs*	D112	[60]
	E90	E90A	α1	*Sc*	D112	[58]
	E93	E93A	α1	*Sc*	D115	[58]
	D115	D115A	α1-β1 Loop	*Hs*	D115	[60]
	E121	E121A	α1-β1 Loop	*Hs*	E121	[60]
	D122	D122A/Y	β1	*Hs*	D122	[56,57,60,61]
	D124	D124A	β1	*Hs*	D124	[60]
	D101	D101A	β1	*Sc*	D124	[58]
	E103	E103A	β1	*Sc*	S126	[58]
NFS Binding	D104	*1	α1	*Hs*	D104	[17]
	E108	*1	α1	*Hs*	E108	[19]
	E111	*1	α1	*Hs*	E111	[19]
	E121-Y123	*2	α1-β1 Loop	*Hs*	E121-Y123	[19]
	D124	D124A/K	β1	*Hs*	D124	[19,62]
	G128	*2	β1	*Hs*	G128	[19]
	V131	*2	β2	*Hs*	V131	[19]
	N146	N146K	β3	*Hs*	N146	[19,56]
	N151	N151A	β3-β4 Loop	*Hs*	N151	[19]
	W155	W155R	β4	*Hs*	W155	[19,56,61]
	Y175	*2	β6	*Hs*	Y175	[19]
	H177	*2	β6-α2 Loop	*Hs*	H177	[19]
ISCU Binding	T142	*2	β3	*Hs*	T142	[19]
	V120	*1	β3	*Sc*	V144	[28]
	V144	*2	β3	*Hs*	V144	[19]
	N146	*2	β3	*Hs*	N146	[19]
	N122	N122A/K	β3	*Sc*	N146	[27]
	Q124	Q124A	β3	*Sc*	Q148	[27]
	T149	*1	β3-β4 Loop	*Hs*	T149	[17]
	P150	*2	β3-β4 Loop	*Hs*	P150	[19]
	N151	N151A	β3-β4 Loop	*Hs*	N151	[19]
	N127	*1	β3-β4 Loop	*Sc*	N151	[28]
	Q153	*1	β4	*Hs*	Q153	[17]
	Q129	Q129A	β3-β4 Loop	*Sc*	Q153	[63]
	W155	W155R/A/F	β4	*Hs*	W155	[61,64]
	W131	W131A/F	β4	*Sc*	W155	[63]
	S157	*1	β4	*Hs*	S157	[17]
	P163	P163G	β5	*Hs*	P163	[19]
	R165	R165C	β5	*Hs*	R165	[19]
	R141	R141A	β5	*Sc*	R165	[63]
Stability	L106	L106S	α1	*Hs*	L106	[65]
	D86	D86A	α1	*Sc*	E108	[66]
	F109	F109L	α1	*Hs*	F109	[67]
	E93	E93A	α1	*Sc*	D115	[66]
	D122	D122Y	α1-β1 Loop	*Hs*	D122	[66]
	Y123	Y123S	α1-β1 Loop	*Hs*	Y123	[67]
	D101	101A	β1	*Sc*	D124	[66]
	E103	103A	β1	*Sc*	S126	[66]
	G130	G130Y	β1	*Hs*	G130	[66]
	T110A	T110A	β2	*Sc*	V134	[63]
	G137	G137V	β2- β3 Loop	*Hs*	G137	[68]
	T118A	T118A	β3	*Sc*	T142	[63]
	V120	V120A	β3	*Sc*	V144	[33]
	Q129A	Q129A	β3-β4 Loop	*Sc*	Q153	[63]
	I130	I130A	β4	*Sc*	I154	[63]
	I154	I154F	β4	*Hs*	I154	[66]
	W155	W155R	β4	*Hs*	W155	[66]
	W131	W131A/F	β4	*Sc*	W155	[63]
	L132	L132A	β4	*Sc*	L156	[63]
	S161	S161I	β4- β5 Loop	*Hs*	S161	[67]
	W173	W173G	β6	*Hs*	W173	[69]
	S181	S181F	α2	*Hs*	S181	[67]
	L182	L182F	α2	*Hs*	L182	[70]
	L185-L186	*3	α2	*Hs*	L185-L186	[70]
	L190	*3	α2	*Hs*	L190	[70]
	L194	*3	α2	*Hs*	L194	[70]
	T196-K197	Truncation	C-Termini	*Hs*	T196-K197	[70]
	L198	L198R/A/C	C-Termini	*Hs*	L198	[71]
	D199	Truncation	C-Termini	*Hs*	D199	[70]
	L200	L200C	C-Termini	*Hs*	L200	[71]
	S201-A210	Truncation	C-Termini	*Hs*	S201-A210	[70]

*1 Chemical shifts observed by NMR. *2 Cryo-EM confirmed predictions from crosslinking, SAXS and NMR. *3 Modeling.

**Table 3:** Notable ISCU Residues.

Function	Residue	Mutation	Location on Protein	Organism	*H sapien* Equivalent	Reference
Iron Binding	C69	C69A/S	β1–β2 Loop	*Hs*	C69	[74,75]
	C95	C95A/S	α2	*Hs*	C95	[74,75]
	H137	*1	α4	*Hs*	H137	[74,75]
	C138	C138A/S	α4	*Hs*	C138	[74,75]
Active Site	C69	C69A/S	β1–β2 Loop	*Hs*	C69	[24]
	D71	D71A, D71V	β2	*Sc, Hs*	D71	[76]
	C95	C95A/S	α2	*Hs*	C95	[24]
	H137	*1	α4	*Hs*	H137	[24]
	C138	C138A/S	α4	*Hs*	C138	[24]
NFS Binding	Y35	Y3F/H/W	N-terminus	*Ec*	Y35	[77]
	L63	L63A	β1	*Sc*	L63	[78]
	A66	*3	β1–β2 Loop	*Hs*	A66	[16,18]
	P67	*3	β1–β2 Loop	*Hs*	P67	[16,18]
	A68	*3	β1–β2 Loop	*Hs*	A68	[16,18]
	C69	C69A/S	β1–β2 Loop	*Hs*	C69	[16]
	D71	D71A	β2	*Sc*	D71	[16,17]
	V72	V72A	β2	*Sc*	V72	[78]
	F94	F94A	β3-α2 Loop	*Sc*	F93	[78]
	C95	C95A/S	α2	*Hs*	C95	[16]
	H137	*1	α4	*Hs*	H137	[16]
	C138	C138A/S	α4	*Hs*	C138	[16]
	M138	M138I	α4	*Sc*	M140	[48]
FXN Binding	A66	*2	β1–β2 Loop	*Hs*	A66	[19]
	P67	*2	β1–β2 Loop	*Hs*	P67	[19]
	A68	*2	β1–β2 Loop	*Hs*	A68	[19]
	C69	*2	β1–β2 Loop	*Hs*	C69	[19]
	G70	*2	β1–β2 Loop	*Hs*	G70	[19]
	D71	*2	β1–β2 Loop	*Hs*	D71	[19]
	L131	*3	α4	*Hs*	L131	[19]
	P132	*3	α4	*Hs*	P132	[19]
	P133	*3	α4	*Hs*	P133	[19]
	V134	*3	α4	*Hs*	V134	[19]
	K135	*3	α4	*Hs*	K135	[19]
	L136	*2	α4	*Hs*	L136	[19]
	H137	*2	α4	*Hs*	H137	[19]
	C138	*2	α4	*Hs*	C138	[19]
	S139	*2	α4	*Hs*	S139	[19]
	M140	*2	α4	*HS*	M140	[16,17]
Stability	C69	C69A/S, *1	β1–β2 Loop	*Hs*	C69	[79]
	D71	D71A/V, *1	β2	*Sc*	D71	[79]
	C95	C95A/S, *1	α2	*Hs*	C95	[79]
	W108	*1	α2	*Hs*	W108	[32]
	H137	*1	α4	*Hs*	H137	[79]
	E144	E144A	α4	*Sc*	E143	[80]
	E144Δ	Truncation	α4	*Sc*	E143	[81]
	D145	D145A	α4	*Sc*	D144	[80]

*1 Chemical shifts observed by NMR. *2 Cryo-EM confirmed predictions from crosslinking, SAXS, and NMR. *3 Modeling.

## Data Availability

Not applicable.

## Supplementary Materials

The following are available online at https://www.mdpi.com/article/10.3390/ijms22116006/s1, Table S1: Complete list of notable frataxin residues targeted for characterization.
