# Supplementary material for: Molecular Details of the Frataxin–Scaffold Interaction during Mitochondrial Fe–S Cluster Assembly
Source: Int J Mol Sci. 2021 Jun 2;22(11):6006. doi: 10.3390/ijms22116006 (PMC8199681; doi:10.3390/ijms22116006)
Supplement: Supplementary file 1 [file ijms-22-06006-s001.zip › ijms-1236373-supplementary.pdf]

Supplementary Table S1.

Function	Residue	Mutation	Location on Protein	Organism	Hs Equivalent	Reference
Iron Binding	E92	E92A	α1	*Hs*	E92	[54]
	E96	E96A	α1	*Hs*	E96	[54]
	E100	E100A	α1	*Hs*	E100	[54]
	E101	E101A	α1	*Hs*	E101	[54]
	D104	D104A	α1	*Hs*	D104	[54]
	S105	*1	α1	*Hs*	S105	[16]
	H83	*1	α1	*Sc*	S105	[33]
	A107	*1	α1	*Hs*	A107	[16]
	D86	D86A	α1	*Sc*	E108	[33]
	E108	E108A	α1	*Hs*	E108	[16]
	F109-F110	*1	α1	*Hs*	F109-F110	[16]
	E111	E111A	α1	*Hs*	E111	[53]
	D112	D112A	α1	*Hs*	D112	[82]
	E90	E90A	α1	*Sc*	D112	[33]
	L91	*1	α1	*Sc*	L113	[34]
	L113-A114	*1	α1	*Hs*	L113-A114	[16]
	E93	E93A	α1	*Sc*	D115	[33]
	D115	D115A	α1-β1 Loop	*Hs*	D115	[82]
	A94	*1	α1	*Sc*	K116	[33]
	K116	*1	α1-β1 Loop	*Hs*	K116	[16]
	H95	*1	α1	*Sc*	P117	[33]
	E121	E121A	α1-β1 Loop	*Hs*	E121	[82]
	D122	D122A/Y	β1	*Hs*	D122	[82] [54]
	Y123	*1	β1	*Hs*	Y123	[16]
	D124	D124A	β1	*Hs*	D124	[82]
	D101	D101A	β1	*Sc*	D124	[33]
	V102	*1	β1	*Sc*	V125	[18]
	V125-F127	*1	β1	*Hs*	V125-F127	[16]
	E103	E103A	β1	*Sc*	S126	[33]
	L104	*1	β1	*Sc*	F127	[33]
	S105	*1	β1	*Sc*	G128	[33]
	G130	G130V	β1	*Hs*	G130	[54]
	I154	I154F	β4	*Hs*	I154	[54]
	W155	W155R	β4	*Hs*	W155	[54]
	N140	*1	β4-β5 Loop	*Sc*	K164	[33]
NFS Binding	D104-S105	*1	α1	*Hs*	D104-S105	[16]
	E108-F109	*1	α1	*Hs*	E108-F109	[18]
	F110	*1	α1	*Hs*	F110	[16]
	E111	*1	α1	*Hs*	E111	[18]
	D112-A114	*1	α1	*Hs*	D112-A114	[16]
	D115-T119	*1	α1-β1 Loop	*Hs*	D115-T119	[16]
	E121-Y123	*2	α1-β1 Loop	*Hs*	E121-Y123	[18]
	D124	D124A/K	β1	*Hs*	D124	[18,55]
	V125-F127	*1	β1	*Hs*	V125-F127	[16]
	G128	*2	β1	*Hs*	G128	[18]
	V131	*2	β2	*Hs*	V131	[18]
	N146	N146K	β3	*Hs*	N146	[18]
	K147	*4	β3	*Hs*	K147	[86]
	N151	N151A	β3-β4 Loop	*Hs*	N151	[18]
	K152	*4	β3-β4 Loop	*Hs*	K152	[86]
	I154	I154F	β4	*Hs*	I154	[18], 54]
	W155	W155R	β4	*Hs*	W155	[18] [54]
	K171-N172	*1	β5-β6 Loop	*Hs*	K171-N172	[16]
	W149	W149G	β6	*Sc*	W173	[35]
	Y175	*2	β6	*Hs*	Y175	[18]
	H177	*2	β6-α2 Loop	*Hs*	H177	[18]
	A204	*1	C-Termini	*Hs*	A204	[16]

ISCU Binding	G107	*1	β1-β2 Loop	*Sc*	G130	[18]
	T110	*1	β2	*Sc*	V134	[18]
	L111	*1	β2	*Sc*	K135	[18]
	T142	*2	β3	*Hs*	T142	[18]
	Y119	*1	β3	*Sc*	Y143	[18]
	V120	*1	β3	*Sc*	V144	[38]
	V144	*2	β3	*Hs*	V144	[18]
	N122	N122A/K	β3	*Sc*	N146	[42]
	I145-K147	*1	β3	*Hs*	I145-K147	[16]
	K123	K123T	β3	*Sc*	K147	[42]
	Q124	Q124A	β3	*Sc*	Q148	[42]
	T149	*1	β3-β4 Loop	*Hs*	T149	[16]
	P150	*2	β3-β4 Loop	*Hs*	P150	[18]
	N151	N151A	β3-β4 Loop	*Hs*	N151	[18]
	N127	*1	β3-β4 Loop	*Sc*	N151	[34]
	K152	*1	β3-β4 Loop	*Hs*	K152	[16]
	Q153	*1	β4	*Hs*	Q153	[16]
	Q129	Q129A	β3-β4 Loop	*Sc*	Q153	[32]
	I154	I154F	β4	*Hs*	I154	[54]
	W155	W155R/A/F	β4	*Hs*	W155	[54,77]
	W131	W131A/F	β4	*Sc*	W155	[32]
	L156-S158	*1	β4	*Hs*	L156-S158	[16]
	P163	P163G	β5	*Hs*	P163	[18]
	K164	*1	β5	*Hs*	K164	[16]
	R165	R165C	β5	*Hs*	R165	[18]
	R141	R141A	β5	*Sc*	R165	[32]
	W168	*1	β5	*Hs*	W168	[16]
	N154	*1	β6- β7 Loop	*Sc*	D178	[34]
	V166	*1	α2	*Sc*	L190	[34]
	I170	*1	α2	*Sc*	L194	[34]
Stability	L106	L106S	α1	*Hs*	L106	[60]
	D86	D86A	α1	*Sc*	E108	[33]
	F109	F109L	α1	*Hs*	F109	[61]
	E93	E93A	α1	*Sc*	D115	[33]
	D122	D122Y	B1	*Hs*	D122	[54]
	Y123	Y123S	α1-β1 Loop	*Hs*	Y123	[61]
	D101	101A	β1	*Sc*	D124	[33]
	E103	103A	β1	*Sc*	S126	[33]
	G130	G130Y	β1	*Hs*	G130	[54]
	T110A	T110A	β2	*Sc*	V134	[32]
	G137	G137V	β2- β3 Loop	*Hs*	G137	[84]
	T118A	T118A	β3	*Sc*	T142	[32]
	V120	V120A	β3	*Sc*	V144	[32]
	Q129A	Q129A	β3-β4 Loop	*Sc*	Q153	[32]
	I130	I130A	β4	*Sc*	I154	[32]
	I154	I154F	β4	*Hs*	I154	[54]
	W155	W155R	β4	*Hs*	W155	[54]
	W131	W131A/F	β4	*Sc*	W155	[32]
	L132	L132A	β4	*Sc*	L156	[32]
	S161	S161I	β4- β5 Loop	*Hs*	S161	[61]
	W173	W173G	β6	*Hs*	W173	[85]
	S181	S181F	α2	*Hs*	S181	[61]
	L182	L182F	α2	*Hs*	L182	[56]
	L185-L186	*3	α2	*Hs*	L185-L186	[56]
	L190	*3	α2	*Hs*	L190	[56]
	L194	*3	α2	*Hs*	L194	[56]
	T196-K197	Truncation	C-Termini	*Hs*	T196-K197	[56]
	L198	L198R/A/C	C-Termini	*Hs*	L198	[57]
	D199	Truncation	C-Termini	*Hs*	D199	[56]
	L200	L200C	C-Termini	*Hs*	L200	[57]

S201-A210	Truncation	C-Termini	*Hs*	S201-A210	[56]

*1 Chemical shifts observed by NMR
*2 Cryo-EM confirmed predictions from crosslinking, SAXS and NMR
*3 Modeling
*4 LC-MS/MS Analysis
